# The Correlates of Leisure Time Physical Activity among an Adults Population from Southern Taiwan

**DOI:** 10.1186/1471-2458-11-427

**Published:** 2011-06-03

**Authors:** Yi-Ju Chen, Ying-Hsiang Huang, Feng-Hwa Lu, Jin-Shang Wu, Linda L Lin, Chih-Jen Chang, Yi-Ching Yang

**Affiliations:** 1 Graduate Institute of Physical Education, Health & Leisure Studies, National Cheng Kung University, Taiwan, Republic of China; 2 Department of Family Medicine, National Cheng Kung University Hospital, Taiwan, Republic of China; 3 Department of Family Medicine, College of Medicine, National Cheng Kung University, No.138, Sheng Li Road, Tainan, Taiwan 70403, Republic of China

## Abstract

**Background:**

Assessing the correlates of practicing physical activity during leisure time is important with regard to planning and designing public health strategies to increase beneficial behaviors among adult populations. Although the importance of leisure time physical activity (LTPA) is highlighted in many Western countries, there are not many publications on physical activity patterns, and even less on their correlates, in non-Western societies. The goal of this study was thus to explore the determinants influencing adults' leisure time physical activity (LTPA) in a city in southern Taiwan.

**Methods:**

A cross-sectional population-based study was conducted in 2007, using a standardized questionnaire. Energy expenditure was dichotomized into two groups based on the recommended levels of moderate physical activity from LTPA: ≥10 or < 10 MET·hr·wk^-1^. Logistic regression analyses were applied to the results.

**Results:**

A total of 762 subjects with valid data took part in the study (mean age 53.8 ± 13.8 years). In multivariate logistic regression analysis, we found the following results: Age was positively associated with LTPA. Adults with stronger perceived convenience of exercise facilities (OR = 2.04; 95%CI = 1.28-3.24) and past exercise experience in school (OR = 1.86; 95%CI= 1.19-2.91) participated in more LTPA. Subjects with more general social support (OR = 1.66;95%CI = 1.13-2.44), greater knowledge about the health benefits of exercise (OR = 1.85;95%CI = 1.25-2.74), more sports media consumption (OR = 1.94;95%CI = 1.26-2.98), and higher self-efficacy (OR = 3.99;95%CI = 2.67-5.97) were more likely to engage in LTPA. Further analysis comparing different sources of social support showed only social support from friends had a significant positive association (OR = 1.73;95%CI = 1.14-2.63) with increased LTPA.

**Conclusions:**

LTPA in southern city of Taiwan showed some unique associations with age, socioeconomic status and media consumption that are not commonly reported in the Western World and similar associations with regards to psychosocial correlates of LTPA participation. Further studies from developing countries are warranted to highlight culture-specific differences in physical activity participation.

## Background

Regular physical activity has been proven to reduce the incidence and mortality of many chronic diseases such as type 2 diabetes, hypertension, cardiovascular disease, osteoporosis, stroke, and cancer [1-5]. Leisure-time physical activity (LTPA) was listed as one of the ten leading health indicators in the U.S. Healthy People 2010 report [6]. More specifically, the U.S. Center for Disease Control and Prevention (CDC) and the American College of Sports Medicine (ACSM) have recommended that adults should engage in at least 30 min of moderate activity 5 d·wk^-1 ^or 20 min of vigorous activity at least 3 d·wk^-1^[7]. In order to promote non-sedentary environments and encourage more people to become active, it is necessary to know the determinants of adults' physical activity. Studies have shown that the prevalence and correlates of LTPA differ across countries and ethnic groups, and while most works in this field have been done in Western countries [8-10], research on the correlates of physical activity in Asian contexts is relatively scarce [11,12]. This is very important, because culture serves as a basis for the decisions that guide an individual's behavior. In many Asian contexts, the patterns of regular LTPA and their interrelations and associations with individual socioeconomic, lifestyle, environmental factors, and physical/emotional support received, are different from those seen in the West. For example, three-generation households are relatively common in many Asian countries, and thus support from family members might be more important for Asian people than that received from friends or peers. Based on these basic cultural differences and their likely influences on individuals' leisure time activities, the purpose of this study was to explore the determining factors which influence the adults' LTPA in a city in southern Taiwan.

## Methods

For the study population, a stratified systemic cluster sample of households was drawn from defined areas of a city in southern Taiwan. The baseline survey was conducted in 1996[13] with 1,638 participants (> 20 years of age), and then 10 years later (in 2006-2007) 762 persons returned for a follow-up survey. After excluding 135 subjects who had died during the intervening ten-year period, the age-gender distribution of the 756 individuals who did not participate the follow up survey, with 45.6% being male and an mean age of 51.6 (SD:4.8) years, was similar to the 762 individuals who did participate it, 47.1% of whom were male and with an mean age of 53.8 (SD:13.8) years. Furthermore, there was also no significant difference in the distribution of education levels between the two groups. All 762 subjects were interviewed according to a structured questionnaire, which included items related to demographic characteristics, socioeconomic status, past medical history, lifestyle behaviors, involvement in LTPA, and associated factors related to such activities. Informed consent was obtained from all of the study participants, and the Ethical Committee for Human Research at National Cheng Kung University Hospital approved the study protocol.

### Measurement of physical activity

All subjects were asked to report the details of their LTPA in the past year, including categories, duration per session (in minutes), and frequency (sessions per week, weeks per month, and months per year). In total, there were 24 kinds of LTPA in our questionnaire, based on the cultural preferences in Taiwan, including croquet, gardening, fishing, calisthenics, tai-chi, bowling, folk (or aerobic) dancing, volleyball/table tennis, single (or double) badminton/tennis, slow (or fast) bicycling, baseball/softball, slow (or brisk) walking, hiking, mountain climbing, weight training, jogging/running, martial arts, soccer, basketball, rope jumping, and swimming. A metabolic equivalent (MET) value was then assigned to each reported activity according to the compendium produced by Ainsworth et al [14]. For each individual, LTPA energy expenditure in MET-hours per kilogram body weight can be estimated by summing up the energy expenditure of all activities on a weekly basis (MET·hr·wk^-1^).

### Assessment of potential correlates of LTPA

The determinants of adult physical activity had six classes of factors: a) demographic and biological factors; b) psychological, cognitive, and emotional factors; c) behavioral attributes and skills; d) social and cultural factors; and e) physical environmental factors [15]. To be comparable with previous works, we presented the correlates in a similar way, and details of these as follows.

#### Demographic and Biological Factors

*Age: *three groups: young (aged < 45 years), middle aged (aged 45-64 years) and elderly (≧ 65 years). *Socioeconomic status *(SES) was determined by personal occupation and education level using the modified Hollingshead's Two-Factor Index of Social Position [16]. The summed weighted scores were categorized into I~V social classes. We further redefined these five classes as low (Ⅰ & Ⅱ), middle (Ⅲ), and high (Ⅳ & Ⅴ) SES, respectively. *Care-burden: *two groups, "with" or "without" caregiver responsibilities (with regard to children or sick/elderly family members).

*Marital status: *married/cohabiting, widowed/divorced/separated, and single. *Chronic diseases: *Subjects having one of the following diseases, hypertension, diabetes mellitus, cerebrovascular disease and heart disease, were defined as "with chronic disease".

#### Psychological, Cognitive, and Emotional Factors

##### Knowledge of health and exercise

Knowledge about the benefits of regular and moderate physical activity was measured through two questions with a total of 30 items, including 19 items on the benefit (e.g., exercise can lower blood pressure, relieve stress, and so on) and 11 items on the disease-prevention effects (e.g. exercise can prevent diabetes mellitus, osteoporosis, etc). Knowledge of one item represented one point, and higher scores thus indicated more knowledge about physical activity. *Self-efficacy: *The latent variable self-efficacy was measured with the modified subscales from the 12-item Self-Efficacy for Exercise Behaviors Scales [17]. This modified instrument consisted of six items using a four-point scale: never, seldom, sometimes and frequently.

#### Behavioral Attributes and Skills

##### Previous participation in school sports or exercise in student life

This scale was adapted from Taylor's study and consisted of four items [18]. The frequency of participation (never, seldom, sometimes and frequently) was recorded for school sports teams, inside-school sports competitions, informal sports group, and sports training lessons other than physical education in school. If answering one or more items as "frequently", the respondent was categorized as belonging to the frequent participation group, and otherwise as to the infrequent group. *Cigarette smoking *habit was defined as smoking at least one cigarette per day for at least six months, and *alcohol consumption *habit was defined as drinking alcoholic beverages at least once per week for at least six months. *Sports media consumption: *This scale consisted of four items about the frequency of watching TV sports programs, reading sports reports in newspapers or online, reading sports magazines, and watching live sports games. Higher scores indicated more consumption of sports media.

#### Physical Environment Factors

##### Home equipments

This instrument consisted of 30 items of home exercise equipment (e.g., weights, bicycle, basketball, and so on) with higher scores indicating more such items at home.

##### Perceived convenience of facilities around home

Using a three-point scale (inconvenient, acceptable, and convenient), we asked about the availability of outdoor facilities within two blocks or a five-minute walk from home, and defined the inconvenient and acceptable responses as the "poor group", and the convenient as the "great group".

#### Social and Cultural Factors

##### Social support

This four-point (never, seldom, sometimes and frequently) scale measured support from family members, friends/peers and medical staff with regards to reminding or encouraging the respondent to exercise, exercising with them, or objecting to the respondent exercising. Higher scores indicated more social support from different subgroups.

### Statistical analyses

Based on the physical activity guidelines recommended by ACSM and AHA for healthy adults[7], the "moderate" level of physical activity nominally indicated meeting any of the following three criteria: at least 30 minutes of moderate-intensity activity (or brisk walking) 5 d·wk^-1 ^; > 20 minutes of vigorous-intensity activity 3 d·wk^-1 ^; or 5 days of any combination of walking, moderate-intensity or vigorous-intensity activities achieving at least 600 MET-minutes·wk^-1^(Bauman et al. 2009), Therefore, we dichotomized LTPA energy expenditure by using a cut-off of 10 MET·hr·wk^-1 ^and applied independent-samples T and chi-square tests to evaluate the differences between the two groups with and without LTPA (≥ 10 MET·hr·wk^-1^). Because of the positive skewness of the knowledge of health and exercise scores, self-efficacy scores, sports media consumption scores, social support scores, and home equipment availability, for practical reasons, we arbitrarily and consistently dichotomized these variables at the upper quartile. Associations with LTPA were evaluated by odds ratios (OR) and 95% confidence intervals (CI) derived from multivariate logistic regression modeling. We put the selected variables stepwise into a regression model, age-sex only at first, then adding other demographic and biological factors into model 2; adding psychological, cognitive, emotional factors and behavioral attributes into model 3; adding physical environmental and social/cultural factors into model 4; and finally replacing the social support index with 3 different sources of support variables in model 5. Statistical analyses were performed using SPSS version 15.0, and all tests were two-tailed.

## Results

A total of 762 subjects with valid and complete data were included, with 359 (47.1%) men and 403 (52.9%) women at a mean age of 53.8 (SD:13.8) years. Approximately 60% of the study participants had attended high school or above. About 21.1% of subjects had a smoking history and 14.0% had a drinking history. The mean energy expenditure of LTPA during the one year preceding the interview was 15.5 MET·hr·wk^-1^, and 46.1% of the subjects met the guideline recommendation (≥10 MET·hr·wk^-1^). Table 1 shows the comparisons of socio-demographic characteristics and correlates of LTPA between the two groups with different levels of LTPA. Subjects whose LTPA did not meet the recommended guideline account for a larger percentage in the young adults group, and the low LTPA individuals were more likely to have a care burden, be single and without chronic disease, have infrequent participation in school sports or other exercise programs, and poor perceived convenience of facilities around their home than those who met the recommendation. In addition, participants who did not meet the recommended LTPA had lower self-efficacy scores, less sports media consumption, and less social support.

**Table 1:** The characteristics of two groups with/without regular leisure time physical activity (≥ 10 MET·hr·wk^-1^) ^b ^among adults population from Southern Taiwan, 2007

Variables ^a^	< 10 MET·hr·wk^-1^	≥ 10 MET·hr·wk^-1^	p value
	n = 411 (53.9)	n = 351 (46.1)	
Age			***
< 45	155 (37.7)	56 (16.0)	
45-64	189 (46.0)	173 (49.3)	
≥ 65	67 (16.3)	122 (34.8)	
Gender			
Male	185 (45.0)	174 (49.6)	
Female	226 (55.0)	177 (50.4)	
Socioeconomic status			
Low	270 (65.9)	257 (73.2)	
Middle	82 (20.0)	49 (14.0)	
High	58 (14.1)	45 (12.8)	
Care burden			**
With	96 (23.4)	52 (14.8)	
Without	315 (76.6)	299 (85.2)	
Marital status			**
Married/Cohabiting	318 (77.4)	291 (82.9)	
Widowed/Divorced/Separated	37 (9.0)	41 (11.7)	
Single	56 (13.6)	19 (5.4)	
Chronic disease			**
With	119 (29)	138 (39.3)	
Without	292 (71)	213 (60.7)	
Alcohol consumption			
Yes	63 (15.4)	44 (12.5)	
No	346 (84.6)	307 (87.5)	
Cigarette smoking			
Yes	95 (23.2)	66 (18.9)	
No	314 (76.8)	284 (81.1)	
School sports or exercise program			**
Frequent	60 (14.6)	82 (23.4)	
Infrequent	351 (85.4)	269 (76.6)	
Knowledge of health and exercise scores	7.7 (6.2)	8.5 (6.3)	
Self efficacy scores	3.5 (4.4)	8.1 (5.4)	***
Sports media usage scores	3.2 (2.9)	3.8 (2.9)	**
General social support scores	16.2 (6.6)	17.9 (6.9)	***
Social support from family	7.1 (3.2)	7.6 (3.4)	
Social support from friends	6.2 (3.2)	7.0 (3.5)	**
Social support from medical staff	2.8 (2.6)	3.3 (2.6)	*
Home equipment numbers	3.4 (3.1)	3.9 (3.3)	
Perceived convenience of facilities around home			***
Great	304 (74.1)	309 (88.3)	
Poor	106 (25.9)	41 (11.7)	

Data expressed as Mean (SD) or No. of subjects (percent)

Using independent-samples T test or Chi-square tests, *** p < 0.001; ** p < 0.01; * p < 0.05

^a ^Variables are defined as follows. (1) Care burden. Bearing caregiver responsibilities (family or children): "with" or "without". (2) Chronic diseases include hypertension, diabetes mellitus, cerebrovascular disease, and heart disease: "with at least one" or "without". (3) *Socio-economic status *was determined from the modified *Hollingshead's Two Factor Index of Social Position*. The summed weighted scores were broken down into five social classes. The 1^st ^& 2^nd^; 3^rd^; 4^th ^& 5^th ^classes were categorized as low, middle, and high socioeconomic status respectively. (4) Smoking was defined as having at least one cigarette per day for at least six months. (5) Alcohol consumption was defined as drinking alcoholic beverages at least once a week for six months or more. (6) School sports or exercise programs include school sport teams, inside-school sports competitions, informal sports teams, and specific sports lessons: "frequent"--frequently in one or more items; or "infrequent"--the others. (7) Knowledge of health and exercise includes 19 items about the benefits (e.g., exercise can lower blood pressure, provide sounder sleep, relieve stress, etc) and 11 items about disease-prevention (e.g., exercise can prevent diabetes mellitus, hypertension, etc). (8) Self-efficacy items include the following factors: lack of energy (too tired); lack of time due to occupation; lack of time due to housework or family responsibilities; lack of time due to some social activities; bad weather; lack of a partner. (9) Sport media usage items include watching sports TV programs, reading sports reports in newspaper or on the internet, reading sports magazines and watching live sports game. (10) General social support scores include support from family, friends, or medical staff. Score items include being reminded, or encouraged to exercise, exercising with someone or having someone objected to the respondent exercising. (11) Home exercise equipment consists of 30 items. (12) Perceived convenience of facilities around home: "inconvenient" and "acceptable"--"poor"; "convenient"--"great". ^b ^Based on the recommended levels for moderate physical activity (at least 30 min of moderate activity 5 d·wk^-1 ^or 20 min of vigorous activity at least 3 d·wk^-1^) (Centers for Disease Control and Prevention, 2005)

Table 2 shows the results of the five multivariate logistic regression models. Age was positively associated with LTPA in all the models. Subjects with care burden had less regular LTPA in model 2, but no longer statistically significant in models 3 to 5. Adults with stronger perceived convenience of exercise facilities and past exercise experience at school participated in more LTPA. Subjects with higher general social support, greater knowledge about the health benefits of exercise, more sports media consumption, and higher self-efficacy were more likely to participate in LTPA. Further analysis of the impact of different aspects of social support in model 5 revealed that only support from friends/peers had a significant positive association with increased LTPA.

**Table 2:** Association between regular leisure-time physical activity (≥10 MET·hr·wk^-^^1 ^vs. < 10 MET·hr·wk^-^^1^) and demographic, biological, behavioral, environmental, psychosocial, and cultural factors using multivariate logistic regression analysis in an adult population from southern Taiwan, 2007

**Variables**	**Model 1**	**Model 2**	**Model 3**	**Model 4**	**Model 5**
***Demographic and biological factors***					
Sex					
Female	1 (ref.)	1 (ref.)	1 (ref.)	1 (ref.)	1 (ref.)
Male	1.245 (0.92-1.68)	1.17 (0.86-1.61)	1.31 (0.88-1.95)	1.34 (0.89-2.02)	1.36 (0.90-2.04)
Age					
< 45	1 (ref.)	1 (ref.)	1 (ref.)	1 (ref.)	1 (ref.)
45-64	2.59 (1.79-3.76)**	2.62 (1.69-4.05)**	3.08 (1.91-4.95)**	2.68 (1.64-4.36)**	2.63 (1.61-4.30)*
≥65	5.13 (3.33-7.89)**	5.67 (3.26-9.87)**	6.32 (3.43-11.65)**	5.97 (3.21-11.13)**	5.85 (3.14-10.91)*
Socioeconomic status					
High		1 (ref.)	1 (ref.)	1 (ref.)	1 (ref.)
Middle		0.70 (0.40-1.23)	0.77 (0.42-1.41)	0.77 (0.41-1.44)	0.82 (0.44-1.53)
Low		0.59 (0.36-0.98)*	0.91 (0.52-1.59)	0.92 (0.52-1.64)	0.93 (0.53-1.66)
Marital status					
Married/Cohabiting		1 (ref.)	1 (ref.)	1 (ref.)	1 (ref.)
Widowed/Divorced/Separated		0.87 (0.51-1.46)	0.84 (0.48-1.46)	0.89 (0.51-1.56)	0.82 (0.46-1.44)
Single		0.63 (0.34-1.16)	0.73 (0.39-1.40)	0.90 (0.47-1.75)	0.84 (0.43-1.63)
Care burden					
No		1 (ref.)	1 (ref.)	1 (ref.)	1 (ref.)
Yes		0.66 (0.45-0.98)*	0.72 (0.47-1.10)	0.74 (0.48-1.15)	0.75 (0.48-1.16)
Chronic disease					
No		1 (ref.)	1 (ref.)	1 (ref.)	1 (ref.)
Yes		0.98 (0.69-1.39)	1.07 (0.74-1.55)	0.98 (0.67-1.42)	0.99 (0.68-1.46)
***Psychological, cognitive and emotional factors***					
Knowledge of health and exercise scores ^a^			1.71 (1.17-2.51)**	1.85 (1.25-2.74)**	1.84 (1.24-2.73)*
Self efficacy scores ^a^			4.06 (2.72-6.05)**	3.99 (2.67-5.97)**	3.92 (2.62-5.87)*
***Behavioral attributes and skills***					
School sports or exercise program					
Infrequent			1 (ref.)	1 (ref.)	1 (ref.)
Frequent			1.83 (1.18-2.85)**	1.86 (1.19-2.91)**	1.88 (1.20-2.93)*
Cigarette smoking					
No			1 (ref.)	1 (ref.)	1 (ref.)
Yes			0.68 (0.42-1.08)	0.72 (0.45-1.16)	0.73 (0.45-1.18)
Alcohol consumption					
No			1 (ref.)	1 (ref.)	1 (ref.)
Yes			0.62 (0.36-1.04)	0.62 (0.36-1.05)	0.59 (0.35-1.01)
Sports media use scores ^a^			2.05 (1.34-3.14)**	1.94 (1.26-2.98)**	1.94 (1.26-2.99)*
***Physical environment factors***					
Home equipment numbers ^a^				1.08 (0.72-1.61)	1.09 (0.73-1.63)
Perceived convenience of facilities					
Poor				1 (ref.)	1 (ref.)
Great				2.04 (1.28-3.24)**	2.03 (1.28-3.23)*
***Social and cultural factors***					
General social support scores ^a^				1.66 (1.13-2.44)**	-
Social support from family				-	1.04 (0.69-1.55)
Social support from friends				-	1.73 (1.14-2.63)*
Social support from medical staff				-	1.11 (0.77-1.59)
	*R^2^_CS _*= 0.080,	*R^2^_CS _*= 0.093,	*R^2^_CS _*= 0.166,	*R^2^_CS _*= 0.199,	*R^2^_CS _*= 0.216,
	*R*^*2*^_*N *_= 0.106	*R*^*2*^_*N *_= 0.124	*R*^*2*^_*N *_= 0.222	*R*^*2*^_*N *_= 0.266	*R*^*2*^_*N *_= 0.289

Data were expressed as OR (95% CI); Logistic regression analysis, * p < 0.05; ** p < 0.01; *R*^*2*^_*CS *_= Cox & Snell R Square; *R*^*2*^_*N *_= Negelkerke R Square

^a ^Upper quartile vs. lower three fourth,

## Discussion

In contrast to most previous studies conducted in the United States and Europe, which found that participation in physical activity decreases with age [9,19], we found that age was positively associated with LTPA participation, which is consistent with other studies conducted in Asia[11,12]. This effect is mainly due to increased LTPA participation after retirement [20], which occurs between the ages of 60-65 for most Taiwanese. In addition, another reason might be that young adults do not have much time to exercise because they need to work long hours, take care of small children or elderly, and face considerable economic pressure. In the analysis of self-report perceived barriers to LTPA, we found that long work hours and family responsibilities were the most common responses (data not shown).

In previous reports, indicators of high SES were positively associated with participation in exercise/sports, however, in this study, no significant correlation was found. This is perhaps because most earlier studies assessed SES separately via education, income, or occupation [21,22]. In addition, about 57% of our subjects were categorized as less-skilled workers, and their mean LTPA was 17.4 MET·hr·wk^-1 ^(data not shown), which was higher than the average of the population. Therefore, the respondents tended to participate in LTPA regardless of their socioeconomic status. Moreover, in Sekine's study, it was found that in three populations of civil servants, high SES individuals generally had high control, high demand and long work hours, which might therefore reduce their time available for LTPA [23].

With regard to behavioral attributes and skills, in contrast to previous studies which reported that sport media consumption had a weak or no association with physical activity [15], our subjects with more sports media consumption were more likely to participate in regular LTPA. The possible reason might be that people who have participated regularly in some kinds of sports in Taiwan may have more access to and pay more attention to information regarding these specific activities. Several previous studies demonstrated an equivocal relationship between participation in athletics during high school and college and later involvement in physical activity [24,25]. For example, Vanreusel et al. reported that youth sports' potential contributions to physical activity in adulthood may be attributed to the prolonged socialization process that occurs when adolescents persist in such activities [25]. In the current study, subjects who had frequent previous school sports or exercise program participation were more likely to engage in exercise/sports during adulthood. Therefore, developing a regular habit of physical activity at younger ages may be an effective approach to increasing participation in regular exercise in adulthood.

With regard to psychological, cognitive and emotional factors, self-efficacy was reported as the most consistent correlate of physical activity behavior in adults [26,27]. In a prospective study of 277 university students, a model of the relation between social cognitive variables and physical activity eight weeks later was tested, and the results shown that self-efficacy had the greatest total effect on physical activity [27]. We had similar results, as self-efficacy (OR = 3.99) also had the strongest correlation with LTPA participation. However, the positive association of regular LTPA participation with knowledge related to health and physical activity was found in national survey conducted in Singapore [28], but not shown in a population from an urban primary care center in USA [29]. Our study subjects who had more knowledge about the health benefits of exercise were more likely to participate in LTPA, as in the Singaporean study. Furthermore, for the 30 items about the benefits or disease-prevention effects of regular exercise, the mean scores were about 7.7 and 8.5 for the low and high LTPA participation group, respectively. The low mean score of the former indicates that health care providers, educators, and governments need to make more efforts to promote the health benefits of physical activity.

Among the social and cultural factors influencing physical activity, social support for exercise from family, friends, or medical staff is probably the most clearly established determinant. Social support may occur in various ways, and may be informational, emotional, structural and evaluative. Many studies have shown the importance of social support in enhancing physical activity [29-31]. For example, Sharma et al. found that frequency of social support from friends, but not from family, was a significant predictor of the LTPA of 240 African-American women [30]. In Brownson et al' study, social variables that were associated with physical activity included having friends who encouraged exercise and having at least one friend to exercise with. However, support from relatives did not correlate significantly with greater LTPA participation [31]. Our study showed that only social support from friends/peers had a significant positive association with LTPA, which was in contrast to our initial hypothesis that support from family members is more important in an Asian context. This also reflects how family and social changes in recent decades have significantly altered the family relationships in Taiwan, making them closer to those seen in Western contexts. The practical implications of this is that health educators can make good use of this naturally occurring source of social support, and/or encourage individuals to find exercise partners. They can thus utilize a "buddy system", in which a group of physically active people is paired up with a group physically inactive people to increase the physical activity of the latter.

A visit to a primary healthcare provider appears to be an appropriate fitting time for physical activity counseling, especially due to the fact that the mean number of physician visits per year in Taiwan is about fifteen [32]. In addition, advice about physical activity to ethnic minorities or special populations, such as the poor and underserved, older adults, and those with chronic illness, may be particularly relevant, because there is even greater need for physical activity among such groups [33]. However, in this study, the social support from medical staff did not have a significant association with LTPA. Since lack of time, incentives and counseling skills are the main obstacles with regard to physicians providing effective support to patients. Several studies have evaluated the outcomes of primary care-based physical activity intervention, and recommended the integration of physical activity counseling into routine practice [34]. The national health promotion and disease prevention objectives in the Healthy People 2010 report [35] and set out by the U.S. Preventive Services Task Force [36] recommend that healthcare providers counsel their patients to be physically active as part of their routine healthcare visits, yet the majority of physicians do not do this. The PACE+ (Patient-centered Assessment and Counseling for Exercise and nutrition) program was thus developed as a more effective protocol that incorporates the modifiable determinants of exercise that have proven to be effective in physician-patient physical activity counseling [37].

Considering the impact of home exercise equipment, there was no significant correlation between the amount of such equipment and LTPA. Some studies have reported that the availability of exercise equipment is a convincing environmental determinant of vigorous physical activity/sports participation [38]. In this study, we focused on the total energy expenditure of LTPA, but not on different types of physical activity, and thus the correlation might not be obvious. Also, our study subjects mostly preferred outdoor activities, such as walking, calisthenics, Tai-Chi, and hiking, which are all of mild-moderate intensity and require little equipment.

The majority of public health studies have used self-report surveys to assess people's perceptions of their environments, such as the number of nearby recreational facilities [39]. Hoehner et al. found no direct association between the presence of facilities and meeting recommended physical activity levels [40]. Their results suggested that individual-level factors and other environmental supports besides proximity are thus required. In this study, we measured the perceived convenience of facilities around the home instead of the number of nearby facilities. Because respondents with unfamiliarity with or disinterest in such facilities may include these in the assessment but never use them for recreational purposes, in this regard, perceptions of convenience may be more important than measures of their number. We thus hypothesized that there was an association between the perceived convenience of recreational facilities and their use, and found that adults with stronger perceived convenience of exercise facilities participated in more LTPA. Another explanation is that active respondents might be more likely to perceive recreational facilities as accessible [41]. Research on how elements of the natural and built environment affect physical activity is increasing [42], and a meta-analysis of selected environmental characteristics to explore the relationship between perceived environment and physical activity showed that the perceived presence of PA facilities and the existence of sidewalks were both positively associated with PA [43]. Thus, we should try to develop more comprehensive ecological models that incorporate variables beyond basic demographic information to promote LTPA.

There are some limitations in this study. First, because of the cross-sectional design, we can not infer causal relationships between LTPA and the correlates, and the inaccurate reporting of LTPA status that is inevitably encountered in self-reports may have lead to misobservations of the relationships. Second, during data collection, we did not measure the speed within each category of LTPA. For instance, the jogging/running category may include activities with paces that range from jogging and walking in combination (6 METs) to running at 7.5 mph (12.5 METs). Thus, the MET values for each specific category may not estimate LTPA exactly. Third, we focused on LTPA, but did not fully cover the physical activity involved in transportation, and covered even less about the activity involved in occupations and household work, since the determinants for non-LTPA must be different to those for LTPA. Fourth, we conducted this study in a city, and thus we may not be able to extrapolate the results directly to other populations, particularly adults in rural areas.

## Conclusions

A total of 46.1% of the adults studied in this city in southern Taiwan engaged in the recommended level of LTPA. In addition, the respondents' levels of LTPA were closely associated with psychological, cognitive, and emotional factors; behavioral attributes and skills; social and cultural factors; and physical environment factors. These findings have public health implications with regard to designing strategies to promote participation in leisure time physical activity.

## Competing interests

The authors declare that they have no competing interests.

## Authors' contributions

YHH, JSW, FHL and CJC participated in the background research and the design of the study. YJC performed the statistical analyses and drafted the manuscript. YCY conceived of the study, participated in its design, and coordination, and he helped with the statistical analysis and writing the manuscript. LLL assisted with data analyses. All authors read and approved the final manuscript.

## Pre-publication history

The pre-publication history for this paper can be accessed here:

http://www.biomedcentral.com/1471-2458/11/427/prepub
